# Supplementary material for: Lactobacillus gasseri RW2014 Ameliorates Hyperlipidemia by Modulating Bile Acid Metabolism and Gut Microbiota Composition in Rats
Source: Nutrients. 2022 Nov 22;14(23):4945. doi: 10.3390/nu14234945 (PMC9737415; doi:10.3390/nu14234945)
Supplement: Supplementary file 1 [file nutrients-14-04945-s001.zip › nutrients-1978291-supplementary.pdf]

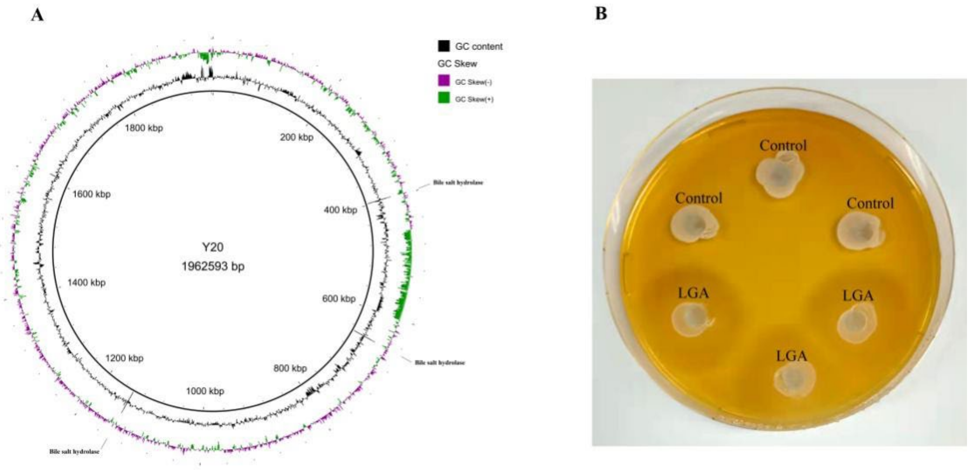

Figure S1. The genome analysis of LGA and the determination of bile salt hydrolase activity. A, LGA genome. B, bile salt hydrolase activity detected in solid medium.

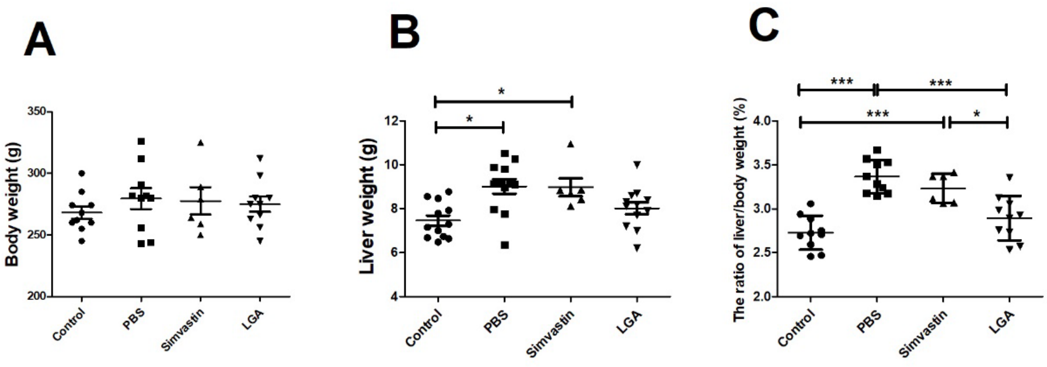

Figure S2. The effect of *L. gasseri* on body weight and liver weight in hyperlipidemia rat. A, the body weight, B, the liver weight of rat. C, the ratio of liver/body weight. The data were analyzed by analysis of variance (ANOVA). $P<0.05$ means the significantly difference. * $P<0.05$, *** $P<0.001$.

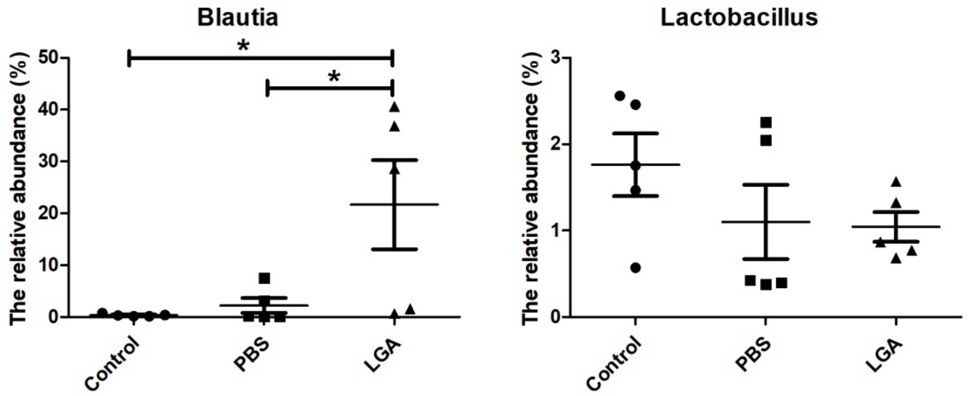

Figure S3 the relative abundance of genera of Blautia and Lactobacillus. A and B represent the Blautia and Lactobacillus genus, respectively. The data were analyzed by analysis of variance

(ANOVA). * P < 0.05, ** P < 0.01, *** P < 0.001.

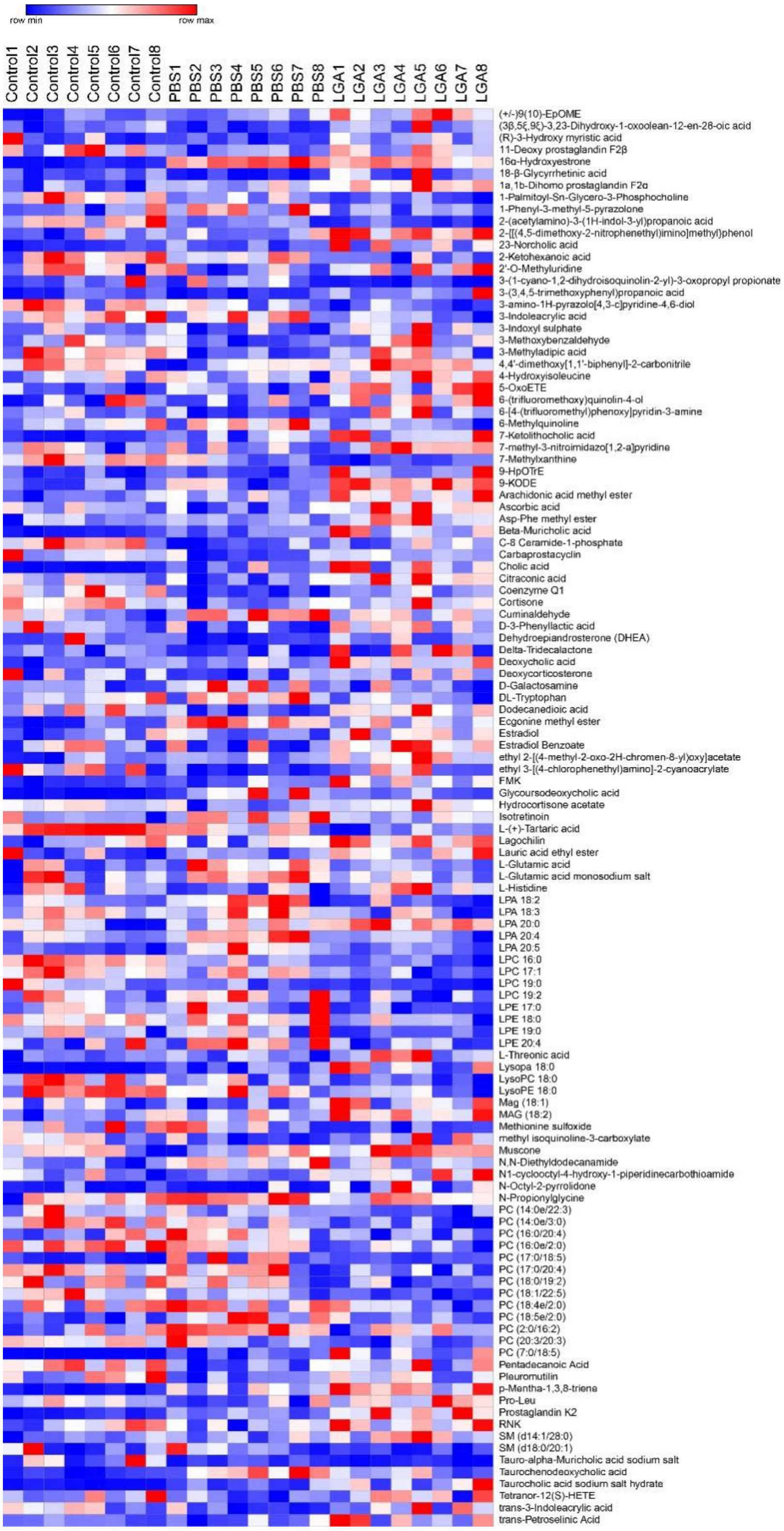

Figure S4. heatmap of non-target metabolism of serum.
